# Annual acknowledgement of reviewers

**DOI:** 10.1186/1471-2318-13-9

**Published:** 2013-02-19

**Authors:** Emily Crow

**Affiliations:** 1 BioMed Central, 236 Grays Inn Rd, WC1X 8HB, London, UK

## Abstract

**Contributing reviewers:**

The editors of *BMC Geriatrics* would like to thank all our reviewers who have contributed to the journal in Volume 12 (2012).

## 

Ivo Abraham

USA

Sharon Andrews

Australia

Kaarin Anstey

Australia

Joshua Armstrong

Canada

Torunn Askim

Norway

Meytal Avgil Tsadok

Canada

Kirsten Avlund

Denmark

Anna Azulai

Canada

Benedetta Bartali

USA

Gillian Bartlett

Canada

Clemens Becker

Germany

Lorne Bellan

Canada

Astrid Bergland

Norway

Louis Bherer

Canada

Astrid Bijlsma

Netherlands

Nienke Bleijenberg

Netherlands

Benoit Boland

Belgium

Mary Bollinger

USA

Veronique Boscart

Canada

Rebecca Boxer

USA

Caroline Brand

Australia

Jackie Bridges

United Kingdom

Christine Brown Wilson

United Kingdom

John Browne

Ireland

Allison Burfield

USA

Helena Burger

Slovenia

Markus A. Busch

Germany

Robert Butler

USA

Bianca Maria Buurman

Netherlands

Mateo Cabre

Spain

Ian Cameron

Australia

John Campbell

New Zealand

Julie Cartwright

USA

Marta Castro

Spain

Tommy Cederholm

Sweden

Steven Cercy

USA

Theodore Cicero

USA

Amber Collins

USA

Fawn Cothran

USA

Jeffrey Craighead

USA

Julia Crilly

Australia

Greta Cummings

Canada

Bjørg Dale

Norway

Thierry Dantoine

France

Carolyn De Coster

Canada

Jan Degryse

Belgium

Anne Dewar

Canada

Mario Di Napoli

Italy

Colleen Doyle

Australia

Paul Drawz

USA

Claudia Drossel

USA

Charlotte Edwardson

United Kingdom

Thorlene Egerton

Norway

E Wesley Ely

USA

Maria Engström

Sweden

Mary Ersek

USA

Sara Espinoza

USA

Ekere Essien

USA

Carol Farran

USA

Lois Finch

Canada

Michael Froehner

Germany

Katsuo Fujiwara

Japan

Svetla Gadzhanova

Australia

Joshua Gagne

USA

Chris Gale

United Kingdom

Xiang Gao

USA

Josep Garre-Olmo

Spain

Claudine Geser

Switzerland

William Gibson

United Kingdom

Margalida Gili

Spain

Adrienne Gilligan

USA

Aaron Gilson

USA

Caroline Glendinning

United Kingdom

Bamini Gopinath

Australia

Beatrix Grubeck-Loebenstein

Austria

Danan Gu

USA

Antonio Guaita

Italy

Jason Guertin

Canada

Mark Haddad

United Kingdom

Andreana Haley

USA

Jan Hamers

Netherlands

Alexandra Hanlon

USA

Tamara Hayes

USA

Helen Hazuda

USA

Tim Henwood

Australia

Carla Hermann

USA

Marion Hetherington

United Kingdom

Anne-Marie Hill

Australia

Keith Hill

Australia

Falk Hoffmann

Germany

Guan-Hua Huang

Taiwan

Martijn Huisman

Netherlands

William Hung

USA

Paulette Hunter

Canada

Kathleen Hunter

Canada

Alvaro Javier Idrovo

Mexico

Jeremy Jacobs

Israel

Carlos Jaramillo

USA

Ulrike Junius-Walker

Germany

Ichiro Kai

Japan

Hulagu Kaptan

Turkey

Marie-Jeanne Kergoat

Canada

Ngaire Kerse

New Zealand

Su Hyun Kim

South Korea

Erika Kobayashi

Japan

Ayumi Kono

Japan

Siran Koroukian

USA

Henk Kraijo

Netherlands

Ed Kuijper

Netherlands

Alexander Kulminski

USA

Jeff Kwong

Canada

Kate Laver

Australia

Ingrid Leiknes

Norway

Carrie Levin

USA

Lene Levy-Storms

USA

Darren Lipnicki

Australia

Gill Livingston

United Kingdom

Pk Loh

Australia

Stephen Lord

Australia

Weixiang Luo

Hong Kong

Alasdair Maclullich

United Kingdom

Anne-Marie Mahoney

Australia

Gordon Mallarkey

United Kingdom

James Malone Lee

United Kingdom

Finbarr Martin

United Kingdom

Margaret Mcgregor

Canada

Audrey Mckinlay

New Zealand

Hongdao Meng

USA

Laura Middleton

Canada

Michelle Miller

Australia

Thais Minett

United Kingdom

Gk Mini

India

Arnold Mitnitski

Canada

Kirsten Moore

Australia

Vincent Mor

USA

Teresa Moreno-Casbas

Spain

Vikky Morris

United Kingdom

Jennifer Morse

USA

Enrico Mossello

Italy

Wendy Moyle

Australia

David Mudge

Australia

Geoff Murray

Australia

Chiara Mussi

Italy

Joseph Mylotte

USA

Maria Nabal

Spain

Masa Narita

USA

Stanton Newman

United Kingdom

Nguyen Dinh Nguyen

Australia

Alessandro Nobili

Italy

Inger Nordin Osson

Sweden

Louise Nygård

Sweden

Thomas Obisesan

USA

Marcel Olde Rikkert

Netherlands

Tone Kristin Omsland

Norway

Graziano Onder

Italy

Adrienne O'Neil

Australia

Hannah O'Rourke

Canada

Robert Paul

USA

Shelley Peacock

Canada

Nancye May Peel

Australia

Subashan Perera

USA

Konrad Pesudovs

Australia

Eugen-Bogdan Petcu

Australia

Charles Phillips

USA

Philayrath Phongsavan

Australia

Peter Pietschmann

Austria

Jacqueline Pugh

USA

Zoran Radovanovic

Serbia

Taina Rantanen

Finland

Matthias Reinhard

Germany

Stefano Rizza

Italy

Andrew Robinson

Australia

Annie Robitaille

Canada

Kenneth Rockwood

Canada

Leocadio Rodriguez-Manas

Spain

Martin Roland

United Kingdom

Monique Rothan-Tondeur

France

Michael Bjørn Russell

Norway

Elisabeth Rydwik

Sweden

Tami Saito

Japan

David Sanders

United Kingdom

Jessica Sautter

USA

Stefan Sävenstedt

Sweden

Lisette Schoonhoven

Netherlands

Geir Selbak

Norway

Melanie Sereny

USA

Aubrey Sheiham

United Kingdom

Catherine Sherrington

Australia

Yea-Ing Shyu

Taiwan

Marc Silverstein

USA

Doungkamol Sindhusake

Australia

Caroline Sirois

Canada

Zbigniew Siudak

Poland

Howard Smith

USA

Stuart Smith

Australia

Oskar H Sommer

Norway

Alonso Soto

Peru

Stephen Sprigle

USA

Paul Stolee

Canada

Rhonda Stuart

Australia

Brendon Stubbs

United Kingdom

Akihiro Sudo

Japan

Theodore Suh

USA

Jennifer Swindle

Canada

Laurence Taconnat

France

Soraia Tahan

Brazil

Paul Takahashi

USA

Rajesh Tampi

USA

Gudrun Theile

Germany

Stephen Thielke

USA

Susie Thomas

Australia

Pao-Feng Tsai

USA

Kirsti Uusi-Rasi

Finland

Hendrik Van Den Bussche

Germany

Nele Van Den Noortgate

Belgium

Eva Van Der Ploeg

Australia

Marie-Claire Van Nes

Belgium

Trevor Van Schooneveld

USA

Stefano Volpato

Italy

Adrian Wagg

United Kingdom

Douglas Fraser Wares

Switzerland

David Weimer

USA

Mandy Wells

United Kingdom

Heather Whitson

USA

Michelle Wien

USA

Elizabeth Wilson

USA

Robert Winningham

USA

Scott Wright

USA

Bei Wu

USA

Qian Li Xue

USA

Yiqing Yang

USA

Sandra Mg Zwakhalen

Netherlands

## Pre-publication history

The pre-publication history for this paper can be accessed here:

http://www.biomedcentral.com/1471-2318/13/9/prepub

